# Exploring Gender Differences in Adolescent Psychiatric Disorders: A Decade of Research

**DOI:** 10.3390/healthcare14020225

**Published:** 2026-01-16

**Authors:** Lidia Ricci, Pasquale Ricci, Angiola Avallone, Monica Calderaro, Giorgia Cafiero, Leonardo Iovino, Rosaria Ferrara

**Affiliations:** 1 Department of Anatomical, Histological, Legal Medicine and Orthopaedic Sciences, Sapienza University of Rome, 00185 Rome, Italygiorgiaelisa.cafiero@uniroma1.it (G.C.);; 2 Department of Life Science, Health, Health Profession, “Link Campus” University of Rome, 00165 Rome, Italy; p.ricci@unilink.it; 3 OISMA Organizzazione Italiana Studio e Monitoraggio Autismo, 00162 Roma, Italy; avallone98angela@gmail.com; 4 Department of International Humanities and Social Sciences, Università degli Studi Internazionali di Roma UNINT, 00147 Roma, Italy; 5 Department of Economic, Legal, Computer, and Motor Sciences “Parthenope” University of Naples, 80133 Naples, Italy

**Keywords:** adolescence, psychiatric disorders, mental health, gender differences

## Abstract

**Highlights:**

**What are the main findings?**

- Adolescence is a developmental phase characterised by profound biological, emotional and social changes and these changes make adolescents particularly vulnerable to the emergence of psychiatric disorders. In this context, gender differences in psychiatric disorders are of increasing clinical interest.
- This review reports on the main gender differences in psychiatric disorders in adolescence. The results demonstrate the need to develop gender-sensitive clinical approaches in psychiatric disorders in order to facilitate prevention, diagnosis and targeted interventions.

**What are the implications of the main findings?**

- It is crucial that mental health professionals adopt a personalised and culturally sensitive approach that takes into account the adolescent’s gender identity and social context. Furthermore, it is necessary that screening, assessment and care protocols are updated to intercept early signs of distress based on gender-specific modes of expression.

**Abstract:**

**Background**: Adolescence is a developmental phase characterised by profound biological, emotional and social changes and these changes make adolescents particularly vulnerable to the emergence of psychiatric disorders. In this context, gender differences in mental health disorders are of increasing clinical interest. **Method:** We conducted a scoping review of the literature regarding gender differences in psychiatric disorders during adolescence. Three databases, PubMed, Web of Science and EBSCO, were used to identify articles published in English from 2015 until 2025. Twenty-one studies fulfilled the inclusion criteria. **Results:** Ten studies deal with mood disorders, with a focus on gender differences in depression and anxiety during adolescence. Two articles analyse eating disorders, highlighting that girls show higher levels of food restriction and body dissatisfaction. Two studies focus on externalising and neurobehavioural disorders, showing a higher prevalence in boys than in girls. Four articles examine self-harm and suicidal behaviour, where girls report higher rates of suicidal ideation and self-harm. Finally, two studies address personality disorders in adolescence, noting a higher incidence of borderline traits and impulsive behaviour among girls. **Conclusions:** Research has revealed gender differences in the onset, frequency and factors associated with psychiatric disorders in adolescence. Understanding these differences is essential for developing prevention strategies, early diagnosis and specific interventions.

## 1. Introduction

Adolescence represents a crucial transition phase between childhood and adulthood and has always been a subject of reflection [1]. It involves significant biological, cognitive, and social changes that affect how individuals feel, think, make decisions, and relate to the world around them [2]. Above all, these changes increase susceptibility to mental health disorders influencing the onset of psychiatric disorders, such as mood disorders, which often occur more frequently in early adolescence [3]. In other psychiatric disorders, such as schizophrenia, it is estimated that up to 18% of individuals experience their first symptoms before the age of 18 [4].

The mental health of adolescents is becoming an increasingly pressing public health concern [5], not only because suicide remains one of the leading causes of death among young people aged 15 to 19, but also because the majority of psychiatric disorders diagnosed in adulthood originate in childhood or adolescence [2].

The gender gap in mental health also tends to become more pronounced during adolescence and gender itself becomes a central element, in which boys begin to construct and internalise models of what it means to be a man or a woman [6]. In this regard, recent investigations show that mental health problems manifest themselves differently between boys and girls: adolescent girls are more prone to experience internalising disorders, such as depression and anxiety [7]. This is probably due to a greater emotional reactivity and sensitivity to social dynamics [8], with the gender gap tending to increase with age [6].

In the context of research, it is essential to distinguish between sex and gender, as both influence biological, psychological and clinical characteristics in different ways. Sex/gender differences are often marked in neurological, psychiatric and neurodevelopmental conditions, making it necessary to clearly define the two concepts [9]. Sex is a biological concept that relates to the genetic, hormonal, anatomical and physiological characteristics of individuals [10]. Gender, on the other hand, is a social and relational construct that encompasses cultural norms, social roles, identities and power relations, which can change over time and in different contexts. It includes gender identity (the way a person perceives themselves), gender as experienced in everyday life, and gender roles, which can be seen as more fluid dimensions or as distinct social categories [11].

Peer relationships in adolescence become a significant source influencing emotional well-being, and girls often show a higher level of sensitivity, more empathy and fear of rejection, all of which can cause internalisation symptoms [12]. In contrast, boys are likely to internalise these emotions when they encounter stress or conflict in peer relationships, which can lead to an intensification of depressive symptoms. Furthermore, they are more likely to experience externalising problems [6], such as oppositional and aggressive behaviour. In developmental age, boys, in particular, present Attention Deficit/Hyperactivity Disorder and Disruptive Disorders more frequently [13].

### Gender Differences and Mental Health

In the literature, some of these gender differences in psychiatric disorders are well established, while others still present complex aspects that need to be further investigated. Thus, although women have higher rates of mental health disorders, it is still unclear whether this gender disparity differs across different dimensions of mental health [14,15,16]. Growing evidence highlights significant gender-based disparities in adolescent mental health, with female adolescents consistently showing a higher vulnerability to internalising disorders compared to their male counterparts. While males are more frequently associated with externalising behaviours such as conduct disorders and substance use, females tend to report greater emotional distress, particularly anxiety, depressive symptoms, and eating disorders [17,18].

Adolescent girls often face heightened pressures related to body image, academic achievement, and social relationships, which may contribute to increased psychological distress. Societal expectations and gender norms can exacerbate the tendency to internalise emotional difficulties, leading to higher rates of mood-related disorders. Furthermore, hormonal changes during puberty, combined with increased sensitivity to interpersonal stressors, can play a significant role in the onset and persistence of these conditions [19,20].

In recent years, the rise in digital technologies and artificial intelligence has introduced new factors that impact adolescent mental health, often in gender-specific ways. For instance, girls are significantly more vulnerable to the negative effects of social media on body image and self-esteem, reporting higher rates of dissatisfaction and anxiety triggered by appearance-focused content [21,22]. Studies show that adolescent females are more likely to internalise social comparisons and experience emotional distress linked to curated online images and peer feedback.

Moreover, recent research has suggested that adolescent girls may form stronger emotional bonds with AI-driven chatbots compared to boys, potentially using these interactions as coping mechanisms for loneliness or emotional dysregulation [23,24]. While such tools can offer temporary relief, they may also reinforce emotional dependency and reduce real-world social engagement, particularly among girls already experiencing psychological distress.

These findings emphasise the need for gender-sensitive prevention and intervention strategies that address the unique psychological challenges faced by adolescent girls, including those emerging in the digital landscape. Recognising and responding to these differences is essential for developing more targeted and effective mental health support during this critical developmental stage

The aim of this scoping review is to consolidate research on gender differences in psychiatric disorders in adolescence, in order to explore these issues and identify models of care that are more sensitive to individual diversity.

## 2. Materials and Methods

The aim of this scoping review was to analyse the main scientific contributions concerning gender differences in psychiatric disorders in adolescence. The scientific articles examined fell within a 10-year research range (2015–2025). The search included three different electronic databases: PUBMED (https://pubmed.ncbi.nlm.nih.gov/, accessed on 30 November 2025),WEBOFSCIENCE (https://www.webofscience.com/, accessed on 30 November 2025) and EBSCO (https://www.ebsco.com/, accessed on 30 November 2025). 

This scoping review followed the PRISMA-ScR (Preferred Reporting Items for Systematic Reviews and Meta-Analyses extension for Scoping Reviews). A detailed overview of the screening and selection process, illustrated by a flow chart compliant with the PRISMA-ScR standard. The PRISMA checklist for this scoping review was provided. The study was not previously registered.

### 2.1. Stage 1: Identify the Research Question

The objective of this exploratory review is to summarise and consolidate research on gender differences in psychiatric disorders in adolescence, in order to further explore these issues and promote the development of care models that are more sensitive to individual diversity.

### 2.2. Stage 2: Search Strategy

The following keywords were used to identify studies relevant to the research questions:

- ‘gender differences in adolescent mental health’;
- ‘adolescent psychopathology and gender differences’;
- ‘adolescent mental health and sex differences’.

With regard to the period covered by the research, only articles published in English in the last ten years (2015–2025) were included.

The databases and search terms were selected based on the main objective of the review, which was to explore gender differences in adolescent psychiatric disorders. These databases and keywords were chosen to comprehensively cover the literature on adolescent mental health.

The initial search strategy (choice of databases, keywords and combination of search terms) was developed by the lead author. The database searches were carried out by two reviewers (L.I. and A.A.), who independently performed the screening process, evaluating titles and abstracts and resolving any discrepancies through discussion with the research team. The data were then organised into tables and compared by cross-referencing the results of the two reviewers to verify and ensure consistency, completeness and the correct selection of all articles relevant to the review.

### 2.3. Stage 3: Inclusion and Exclusion Criteria (Figure 1)

Searches in the three databases yielded a total of 213 articles: 34 from Web of Science, 21 from EBSCO, and 158 from PubMed. Of these, 29 duplicates were removed. Of the remaining 184 studies, 153 were excluded based on their title and abstract because they did not meet the inclusion criteria. Subsequently, 31 studies were found to be eligible for full-text evaluation. After reading the full text, a further 10 articles were excluded, leaving 21 studies eligible for final inclusion in the review.

Inclusion criteria:

- Studies on a sample of adolescents aged between 10 and 19 years;
- Studies on a sample with a diagnosis of a psychiatric disorder;
- Studies on a sample with a standardised and validated assessment of psychopathological symptoms;
- Studies involving an analysis of gender differences in psychiatric disorders;

Exclusion criteria:

- Studies on a sample with an age outside the range 10–19 years;
- Studies that do not report a psychiatric diagnosis or a standardised and validated assessment of psychopathological symptoms;
- Studies that do not consider gender in the analysis of psychiatric disorders;
- Studies that rely on data reported exclusively by parents or caregivers, without directly involving adolescents;

**Figure 1:**
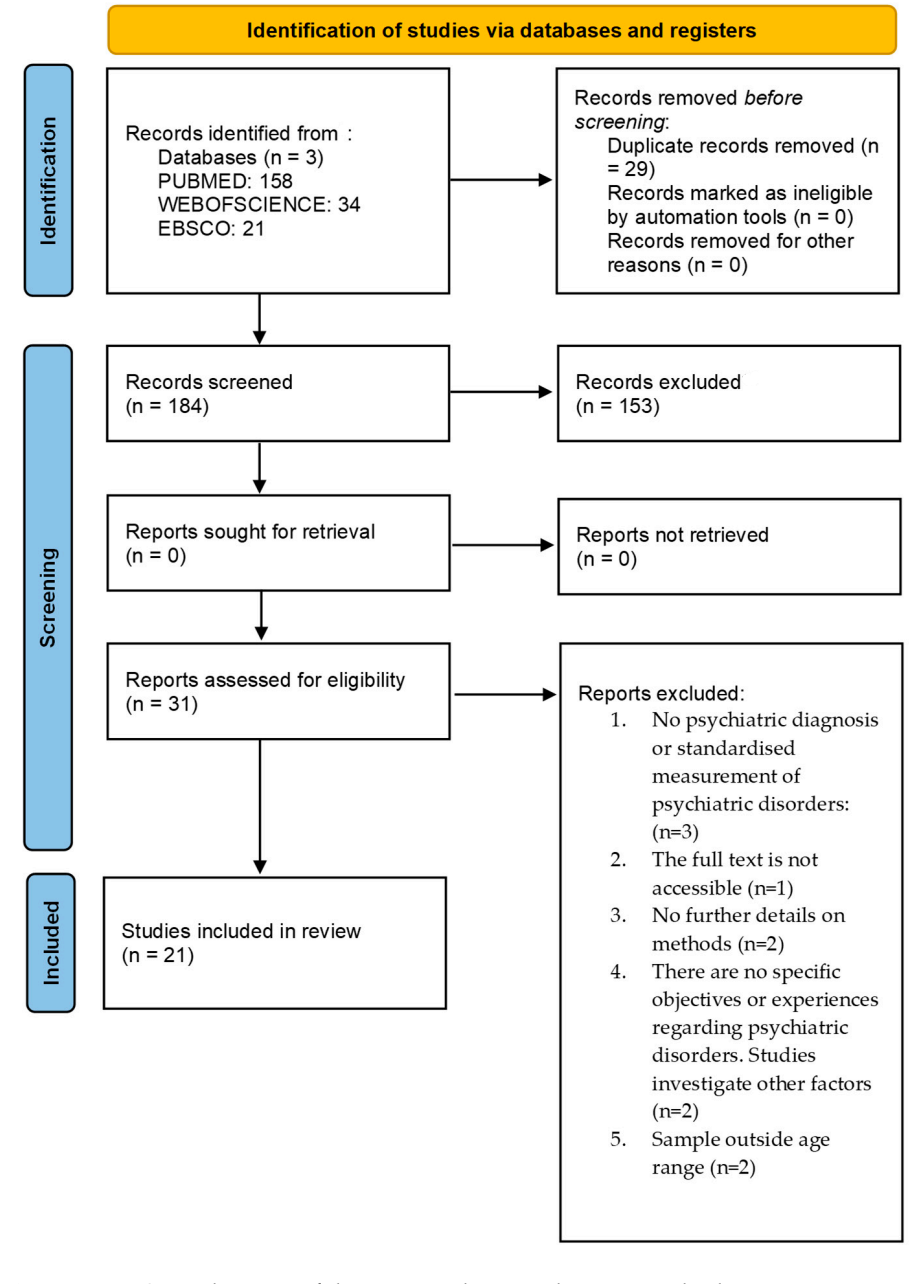
PRISMA diagram of the steps in the record review and selection process.

### 2.4. Detailed Description of Data Recording

At this stage of the process, data extraction was performed, with the information obtained from the selected studies being systematised. Microsoft Excel was used to ensure clarity and consistency. The data collected included: authors, year of publication, study objective, demographic characteristics of the sample and country where the research was conducted, methodological approach used, and finally, the main findings and stated limitations. The tables in the results provide an organised summary of these elements.

### 2.5. Stage 5: Collate, Summarise, and Report Results

In the final phase, the data collected were reorganised according to thematic areas, focusing on concepts relevant to the research question, in line with the exploratory nature of scoping reviews. The information considered most relevant included: the size and characteristics of the sample, the procedures adopted and the main results. The complete set of data is presented in the results section.

## 3. Results

### 3.1. Mood Disorders (Table 1)

A study conducted in Belgium found significantly higher depression, anxiety and self-harm scores among girls than among boys [25]. In addition, another study found that girls reported worse mental health outcomes in four mental health outcomes: life satisfaction, psychological distress, hedonia and eudaimonia compared to boys [6].

**Table 1:** Characteristics of included studies.

Authors/Years	N, Age, Period of Life	Objective	Measures	Specific Findings
Gemma Knowles, Charlotte Gayer-Anderson, Stephanie Beards, Rachel Blakey, Samantha Davis, Katie Lowis, Daniel Stanyon, Aisha Ofori, Alice Turner, Schools Working Group, Vanessa Pinfold, Ioannis Bakolis, Ulrich Reininghaus, Seeromanie Harding, Craig Morgan, 2021 [25]	4353 adolescents (11–14 years)	Estimating the extent and nature of adolescents’ mental health problems	- Self-report Strength and Difficulties Questionnaire (SDQ) - Short Mood and Emotion Questionnaire (SMFQ) - Generalised Anxiety Disorder Scale (GAD-7) - Development and Adolescent Well-being Assessment	The weighted estimate of mental health problems was 18.6%. Each mental health problem was more common among girls than boys
Jun Mo Sunga, Yeon Jung Kim, 2020 [26]	The total number of participants in waves 2, 3 and 4 was 2280 and 2108, respectively (overall mean age about 12.92 years)	Examining gender differences in adolescents’ mental health and the factors influencing them	- Depression subscale of the Korean version of the Symptom Checklist-90-Revised - The scales for attention deficit, aggression and somatic symptoms developed by Cho and Lim (2003) - The social withdrawal scales developed by Kim and Kim (1998) - The Smartphone Addiction Scales developed by Lee et al. (2002) - Korean version of the Conflict Tactics Scale - The school adjustment scales developed by Jung (2009)	Girls show higher scores in depression and somatic symptoms (*p* < 0.001). Boys show a significant prevalence in attention deficit symptoms (*p* < 0.01 and *p* < 0.001).
Sophia M Liles, Anna L Olsavsky, Diane Chen, Connor Grannis, Kristen R Hoskinson, Scott F Leibowitz, Eric E Nelson, Charis J Stanek, John F Strang, Leena Nahata, 2024 [27]	75 adolescents (aged between 12 and 18 years)	Examine the prevalence of internalising symptoms (depression, generalised anxiety, separation anxiety, social anxiety) among transgender/non-binary adolescents	- Children’s Depression Inventory (CDI) - Screen for Child Anxiety Related Emotional Disorders (SCARED) - Child and Adolescent Symptom Inventory-5 (CASI-5)	Most adolescents reported high symptoms of: depression (59 per cent), generalised anxiety (75 per cent), separation anxiety (52 per cent) and social anxiety (78 per cent)
Massimo Apicella, Giulia Serra, Maria Elena Iannoni, Monia Trasolini, Gino Maglio, Elisa Andracchio, Stefano Vicari, 2023 [28]	341 adolescents (aged between 6 and 18 years)	Examining gender differences among adolescents diagnosed with a major depressive episode with mixed characteristics	- Schedule for Affective Disorders and Schizophrenia for School-Age Children-Present and Lifetime Version (K SADS-PL) - Semi-structured diagnostic interview - Children Depression Rating Scale-Revised (CDRS-R) - K-SADS Mania Rating Scale (KMRS) - Child Depression Inventory (CDI) - Clinical Global Assessment Scale (CGAS) - Multidimensional Anxiety Scale for Children (MASC) - Child Behavior Checklist (CBCL) - Columbia Suicide Severity Rating Scale (C-SSRS)	Depression severity was significantly higher in women than in men
Yuchen Li, Jingwen Jiang, Thorhildur Halldorsdottir, Hongru Zhu, Elizabeth Bertone-Johnson, Unnur A Valdimarsdóttir, Xiaobo Zhou, Wei Zhang, Donghao Lu, 2023 [29]	21,239 adolescents (aged 10–19 years)	Exploring the relation ship between premenstrual disorders and gender differences in depressive and anxiety symptoms	- Questionnaire based on theCalendar of Premenstrual Experiences - The 9-item Patient Health Questionnaire (PHQ-9) - 7-item Generalized Anxiety Disorder scale (GAD-7) - Suicidal Behaviors Questionnaire-Revised (SBQ-R)	Premenstrual disorders may contribute to the gender gap in mental health in adolescence
Zheng Zhang, Andi Qiu, Xiangyan Zhang, Yixin Zhao, Lu Yuan, Jing Yi, Qi Zhang, Haidong Liu, Ruoheng Lin, Xiangbin Zhang, 2024 [30]	3769 adolescents (average age: 16.41 ± 1.67 years)	Examining gender differences in psychopathological symptoms	- Middle School Students Mental Health Scale (MSSMHS)	Significant gender differences in psychological symptoms between boys and girls. Depression and anxiety emerged as the main symptoms for boys and girls.
Yeosun Yoon, Mia Eisenstadt, Suzet Tanya Lereya, Jessica Deighton, 2023 [12]	8612 adolescents (aged 11–14 years)	Investigating emerging developmental trends related to gender differences in mental health problems	- Self-report Strengths and Difficulties Questionnaire (SDQ) - Short Warwick and Edinburgh Wellbeing Scale (SWEMWBS)	The girls had lower emotional difficulties and subjective well-being than the boys and both deteriorated over the years.
Evgeniya Yu Privodnova, Nadezhda B Semenova, Olga S Kornienko, Aleksandra V Varshal, Helena R Slobodskaya, 2024 [31]	12,882 adolescents (aged between 11 and 18 years)	Examining gender differences and secular trends in adolescents’ mental health	- Strengths and Difficulties Questionnaire (SDQ)	Significant increase in emotional symptoms and internalising problems (*p* < 0.001) among girls compared to boys
Ingibjorg E Thorisdottir, Bryndis B Asgeirsdottir, Rannveig Sigurvinsdottir, John P Allegrante, Inga D Sigfusdottir, 2017 [32]	The number of participants (age 14–15) in each year was as follows: 2006 *n* = 7232 2009 *n* = 7377 2010 *n* = 7125 2012 *n* = 7202 2014 *n* = 6966 2016 *n* = 7041	Investigating trends in depression and anxiety symptoms among adolescents	- Symptom Check List 90 (SCL-90)	Increase in symptoms compared to previous cohorts: - depression +6.8% girls, +1.6% boys - anxiety +8.6% girls, +1.3% boys
Olympia L K Campbell, David Bann, Praveetha Patalay, 2021 [6]	566,829 adolescents (15 years)	Analysing data for 4 mental health outcomes: psychological distress, life satisfaction, eudaimonia and hedonia in adolescents	- Programme for International Student Assessment (PISA)	Girls have worse mental health than boys, particularly in life satisfaction and psychological distress

Thus, these data confirm a significant prevalence of internalising symptoms, whereas in boys there is a prevalence of externalising symptoms and attention deficit [26].

Another study involved a clinical sample of transgender and non-binary adolescents, helping to highlight differences in menatal health problems, investigating gender differences in particular. 59% of the adolescents reported clinically significant symptoms of depression, while 75% reported clinically significant symptoms of generalised anxiety. In addition, 52% reported borderline separation anxiety and 78% reported borderline social anxiety [27].

Gender differences between males and females also emerge symptomatically. Adolescent girls present depressive symptoms characterised by fatigue, low self-esteem, excessive crying, depressive feelings and suicidal ideation. Boys, on the other hand, show greater psychomotor activation and speech pressure [28].

Another study, however, focused on the role of premenstrual disorders (PMD), which contribute to increased gender differences in anxiety and depressive symptoms. Girls had a higher prevalence of Major Depressive Disorder, Generalised Anxiety Disorder, self-harm and an increased risk of suicide. This prevalence is higher among girls with PMD than among boys and girls without PMD [29].

In contrast to the other studies examined, in an analysis conducted in China, depression, anxiety and paranoia emerged as main symptoms in both boys and girls. In this study, depression emerged as the central factor for adolescent boys, with a strong correlation between depressive symptoms and interpersonal sensitivity. For girls, on the other hand, anxiety emerged as a central factor, closely interconnected with obsessive-compulsive symptoms [30].

Regarding trends in adolescents’ mental health over time, one study examined trends in mental health and psychological difficulties among adolescents, showing a more pronounced worsening of mental well-being and emotional difficulties in girls than in their male peers [12]. A survey in Russia, in particular, found that time trends in all types of mental health problems, with the exception of hyperactivity, differed according to gender, in particular a significant increase in emotional symptoms and internalising problems was found among girls [31]. In Iceland, an analysis showed a significant increase over the study period in the mean symptoms of anxiety and depressed mood for girls. The prevalence of clinically relevant symptoms increased by 6.8% for depression and 8.6% for anxiety in girls [32].

### 3.2. Eating Disorders (Table 2)

Eating disorders (EBD) are characterised by abnormal and health-damaging eating behaviours, such as self-induced vomiting, binge eating, fasting, excessive use of laxatives, extreme exercise and diets to lose weight [33,34].

**Table 2:** Characteristics of included studies.

Authors/Years	N, Age, Period of Life	Objective	Measures	Specific Findings
Yuanyuan Wang, Zhihao Ma, Su Lu, Zhizhou Duan, Amanda Wilson, Yinwei Jia, Yong Yang, Runsen Chen, 2023 [33]	11,440 adolescents (average age 14.74 years)	Investigating eating disorder-related behaviour among adolescents	- Mini International Neuropsychiatric Interview-Criteria for Anorexia Nervosa - Reported Deficits Questionnaire (PDQ-5) - Generalised Anxiety Disorder-7 (GAD-7) - Patient Health Questionnaire-9 (PHQ-9) - 36-Item Short-Form Survey (36-SF)	Male adolescents reported lower anxiety symptoms and a lower likelihood of adopting dietary restrictions than girls
Hannah J White, Emma Haycraft, Deborah J Wallis, Jon Arcelus, Newman Leung, Caroline Meyer, 2015 [34]	527 adolescents (average age 15.9 years)	Examining the Mealtime Emotions Measure for adolescents (MEM-A), gender differences in emotions experienced at mealtimes and levels of eating psychopathology	- Mealtime Emotions Measure-Adolescents (MEM-A) - Eating Disorder Examination Questionnaire (EDE-Q) - Hospital and Anxiety Depression Scale (HADS) - Project-EAT Atmosphere of family meals	Associations between emotional responses to meals and eating psychopathology

In one study, a comparison between males and females on eating disorders showed that adolescent girls had significantly higher scores. Female sexual minority groups (lesbian and bisexual adolescents) showed a higher risk of eating disorders. Among male adolescents, on the other hand, the absence of overt sexual attraction (with heterosexuality as the reference) was associated with a lower likelihood of dietary restriction, elimination behaviour and binge eating. Furthermore, the study found a strong association between DCA symptoms and mental health problems (depression, anxiety and self-harm) in girls compared to boys [33].

In another study, higher levels of anxiety-related negative emotions during family meals also emerged. In particular, girls reported significantly higher levels of mealtime-related anxiety, such as embarrassment, nervousness and anxiety. In contrast, no gender differences were observed for anger or positive emotions during mealtimes between girls and boys. Beyond these gender differences, the study shows strong associations between anxious emotions during mealtimes and increased eating psychopathology in both sexes [34].

### 3.3. Externalising and Neurobehavioural Disorders (Table 3)

One study examined gender differences in mental health among adolescents with substance use problems in outpatient treatment. The results showed that girls had higher rates of depressive and anxiety symptoms than their male peers, while the latter had higher levels of externalising symptoms. Furthermore, it was found that girls had more co-occurring psychiatric disorders than boys [35].

**Table 3:** Characteristics of included studies.

Authors/Years	N, Age, Period of Life	Objective	Measures	Specific Findings
Karin Boson, Mats Anderberg, Johan Melander Hagborg, Peter Wennberg, Mikael Dahlberg, 2022 [35]	455 adolescents (average age 17 years)	Assessing trends in mental distress and associated risk factors after 1 year of treatment for substance use, analysing gender differences.	- Structured interviews - Bivariate analyses - Logistic regressions	Girls had higher levels of symptoms such as anxiety and depression and more frequent access to psychiatric treatment, with depression and suicidal ideation.
Melanie S Askari, Caroline G Rutherford, Pia M Mauro, Noah T Kreski, Katherine M Keyes, 2022 [36]	304,542 adolescents	Estimating the structure of internalising and externalising symptoms and potential temporal dynamics in their association.	- Monitoring the Future (MTF) - Annual cross-sectional surveys	Reduction in externalising symptoms: from −0.06 in 2011 to −0.13 in 2012.

Askari et al. also showed a substantial increase in internalising symptoms among adolescents. In particular, their study showed a decrease in externalising symptoms over time. This relationship between internalising and externalising symptoms appears to have decreased over time first in boys and only later in girls [36].

### 3.4. Self-Injurious Behaviour and Suicidality (Table 4)

A study of adolescents with suicidal behaviour found that the most prevalent personality dimensions for both boys and girls were: Self-humiliating, Introverted, Sad, Inhibited, Oppositional and Borderline Tendency. However, the study also revealed differences between the two sexes, in particular, boys had higher scores in Anxious Feelings, Depressive Affection and Suicidal Tendency. Adolescents, on the other hand, showed higher levels of Delinquency Predisposition and Egocentric traits. In the sample analysed in the study, a history of non-suicidal self-injurious behaviour was present in 49% of the women and 38% of the men [37].

**Table 4:** Characteristics of included studies.

Authors/Years	N, Age, Period of Life	Objective	Measures	Specific Findings
Villar-Cabeza, F., Lombardini, F., Sánchez-Fernández, B., Vila-Grifoll, M., Esnaola-Letemendia, E., Vergé-Muñoz, M., Navarro-Marfisis, M.C., Castellano-Tejedor, C., 2022 [37]	92 adolescents (aged between 13 and 17 years)	Investigates whether gender differences exist among adolescents with suicidal behaviour	- Millon Adolescent Clinical Inventory (MACI)	78% of women were hospitalised for a suicide attempt compared to 58% of men. Half of the women and almost half of the men reported a relational trigger as the cause of their suicidal behaviour.
Raffaela M Flury, Lara Brockhus, Martin Müller, Jonathan Henssler, Aristomenis K Exadaktylos, Jolanta Klukowska-Rötzler, 2022 [38]	612 adolescents (aged between 16 and 18 years)	Analysing gender differences in mental health problems among adolescents	- Swiss Emergency Triage Scale (SETS) - Final diagnosis according to ICD-10	The most frequent diagnoses in adolescents - Reactions to severe stress and adjustment disorders (19.1%); - Alcohol use disorders (17.6%); - Intentional self-harm (17.3%); - Affective disorders (13.7%)
Gabriele Torino, Silvia Leone, Samuele Cortese, Gwen Dieleman, Suzanne Gerritsen, Deborah Maffezzoni, Donato Martella, Rocco Micciolo, Swaran Singh, Cathy Street, Amanda Tuffrey, Leanne Walker, Manuel Zamparini, Giovanni de Girolamo, 2024 [39]	230 adolescents (aged between 16 and 18 years)	Monitor time trends in general health, social functioning, detect gender differences and identify factors associated with Suicidal Thinking and Behaviour (TSI)	- Health of the Nation Outcome Scales for Children and Adolescents (HoNOSCA) - Achenbach’s Empirical Evaluation System (ASEBA) - Assessment of development and well-being (DAWBA) - Transition Readiness and Appropriateness Measure (TRAM)	Females had higher HoNOSCA scores than males (worse physical, behavioural and emotional condition)
Carolyn M Porta, Ryan J Watson, Marion Doull, Marla E Eisenberg, Nathan Grumdahl, Elizabeth Saewyc, 2018 [40]	Adolescents: from a low of 17,278 in 2001 to a high of 19,504 in 2010 (mean age 16.7 years)	Examining emotional distress and suicidal tendencies among male and female students	- Minnesota Student Survey (MSS)	The results reveal a general improvement in youth mental health trends; however, disparities between heterosexual and sexual minority youth have not narrowed over time.

A study was carried out in a Swiss emergency room which showed that girls had higher rates of intentional self-harm, severe stress reaction and adjustment disorders, whereas boys were more likely to present with alcohol-related problems, hyperkinetic disorders and conduct disorders. Furthermore, boys were almost four times less likely to have previous suicide attempts than girls [38].

Another study investigated gender differences in general health and factors associated with Suicidal Thinking and Behaviour (TSI) over time. Girls showed suicidal symptoms at all follow-up times, whereas boys only at 9 months. In addition, study participants reported other comorbid diagnoses, including: neurodevelopmental disorders, mood disorders and anxiety disorders [39].

Significant changes in mental health indicators also occurred in another study. The results showed a decrease over time in the prevalence of emotional distress and suicidal thoughts/attempts, mainly among heterosexual boys and girls. In contrast, the results showed that boys and girls, with partners of both sexes and of the same sex, had a higher risk of sharing indicators of emotional distress and suicidal tendencies at all times studied. Specifically, boys with same-sex partners were almost 4.5 times more likely to ‘feel sad all the time’ and over 3 times more likely to have attempted suicide [40].

### 3.5. Personality Disorders (Table 5)

A study was conducted in Belgium analysing personality disorders during adolescence, with a particular focus on gender differences. The results confirm higher rates of psychopathology in girls, which extend to the level of maladaptive traits. In particular, they present particularly high levels of Emotional Instability and Strangeness. In addition, a higher co-occurrence of psychopathological symptoms and maladaptive traits emerges in girls, with a prevalence of anxious-depressive, angry-irritable and post-traumatic symptoms [41]. Results from another study also indicate that females exhibit higher levels of emotional instability and insecurity, while males show more externalising behaviour. Furthermore, the results suggest that the contribution of narcissistic grandiosity is associated with externalising behaviour. However, when considering gender differences the study found that in adolescent boys and girls, borderline traits and narcissistic vulnerability were found to be more relevant for general psychopathology, whereas narcissistic grandiosity did not have a significant impact [42].

**Table 5:** Characteristics of included studies.

Authors/Years	N, Age, Period of Life	Objective	Measures	Specific Findings
Marie-Céline Gouwy, Lize Verbeke, Kim Dierckx, Lore Van Damme, Olivier Colins, Barbara De Clercq, 2022 [41]	237 adolescents (average age = 15.8 years)	Investigating higher psychopathology scores in girls compared to boys	- The Massachusetts Youth Screening Instrument-Second Version (MAYSI-2) - Dimensional Personality Symptom Itempool (DIPSI)	Results show higher rates of psychopathology in girls than boys, particularly for anxiety-depressive, angry-irritable and post-traumatic symptoms
Ilaria Maria Antonietta Benzi, Andrea Fontana, Rossella Di Pierro, Laura Parolin, Karin Ensink, 2023 (STUDY 1) [42]	725 adolescents (average age 16.22)	Exploring gender differences in psychopathology and maladaptive personality traits	- Borderline Personality Feature Scale for Children-11 (BPFSC-11) - Pathological Narcissism Inventory (PNI)	Girls showed higher scores in borderline traits and general psychopathology; boys showed higher scores in grandiose narcissism
Ilaria Maria Antonietta Benzi, Andrea Fontana, Rossella Di Pierro, Laura Parolin, Karin Ensink, 2023 (STUDY 2) [42]	974 adolescents (average age 16.68)	Examining the structure of gender differences in borderline and narcissistic personality traits	- Youth Self Report (YSR)	Borderline traits were positively associated with general psychopathology, indicating that higher levels of these traits correspond to a higher p-factor

### 3.6. Integrative Summary of Evidence

Analysis of the results reveals a differential pattern of psychopathological expression between genders and in different categories of disorder.

Internalised symptoms (depression, anxiety, emotional difficulties) appear to be consistently more pronounced in girls, while boys more frequently exhibit externalised behaviours such as attention deficit, aggression and behavioural difficulties. Mood disorders and anxiety disorders are more common in adolescent girls, as are eating disorders, which show greater emotional and behavioural associations in females; boys, on the other hand, show higher rates of hyperactivity, impulsivity and externalising symptoms.

Overall, many studies adopt cross-sectional designs, which do not allow for clarification of the evolutionary trajectories of gender differences, and few studies explore the interaction between biological, socio-cultural and contextual factors. Finally, there is a lack of studies from non-Western regions, meaning that many areas are underrepresented.

## 4. Discussion

This scoping review examined the scientific evidence from the last ten years on gender differences in psychiatric disorders in adolescence. The analysis included studies with adolescent samples diagnosed with psychiatric disorders or with a standardised and validated assessment of psychopathological symptoms. The results were reported and divided according to the main diagnostic categories and are in line with the literature. Gender was found to be a determining factor in adolescent mental health, confirming the presence of a differential clinical profile between males and females [26].

- Mood disorders:

The studies included confirm a significant prevalence of depression and anxiety in adolescents. In line with what has been reported in the literature, girls tend to report significantly higher levels of internalisation problems [43], manifesting depressive symptoms more frequently throughout their life cycle [44].

In fact, girls entering the developmental stage before boys undergo the associated physical and hormonal changes [45] and their pubertal development appears to be linked to depressive systems.

Differently than in girls’ depressive symptoms, boys show irritability, behavioural problems, and defiant or isolated behaviour [46,47]

- Eating disorders:

Among the main eating disorders recognised by the Diagnostic and Statistical Manual of Mental Disorders (DSM-5-TR) and the WHO’s International Classification of Diseases and Related Health Problems (ICD-11) are anorexia nervosa, bulimia nervosa, and binge eating disorder [48,49,50].

Our results showed that boys tend to report more positive emotions in response to food stimuli, while girls show higher levels of anxiety during family meals, such as embarrassment, nervousness, and anxiety. This is in line with what has already been highlighted in the scientific literature regarding gender differences in eating disorders.

- Externalising and neurobehavioural disorders:

Externalising disorders include disruptive behaviours, such as those seen in conduct disorder and ADHD. In line with the literature, our findings showed that boys have a higher prevalence of externalising disorders than girls [51]. Externalising disorders often tend to occur concurrently with internalising disorders. This concurrence is associated with worsened psychosocial functioning and increased utilisation of health services [52].

- Self-injurious behaviour and suicidality:

In line with the literature, several studies show that suicidal ideation appears earlier in girls. However, preparatory acts or suicide attempts do not differ by gender in terms of age of onset [53,54,55].

Our findings highlight that in psychiatric emergency settings, girls more frequently present symptoms of self-harm, stress, and adjustment disorders, while boys more often report alcohol abuse and conduct disorders.

One risk factor for self-harm and suicidal behaviour may be emotional dysregulation [56]. In fact, the literature reports an association between perceived limited emotional regulation strategies and suicidal ideation among adolescents [57].

- Personality disorders:

In recent years, there has been growing evidence that personality disorders can manifest as early as adolescence and cause serious disabilities in various areas of life, such as relationships, work, and mental health [58].

The results show gender differences in personality disorders: girls more frequently exhibit emotional instability, anxiety symptoms, and depressive symptoms, while externalising behaviours are more common in boys. These results confirm what has been reported in the literature, which identifies the core of personality disorders in three key elements: identity instability, interpersonal relationship difficulties, and emotional dysregulation [59,60,61].

### 4.1. Barriers to Mental Health Equality: The Role of Gendered Cultural and Social Norms

Despite growing awareness of gender differences in adolescent mental health, several persistent cultural and social barriers continue to hinder the achievement of true parity in psychological well-being between males and females. One of the most prominent factors is the influence of gender role socialisation. From an early age, girls are often encouraged to be emotionally expressive, empathetic, and relationally attuned, which may increase their susceptibility to internalising symptoms such as anxiety, depression, and somatic complaints [17]. In contrast, boys are socialised to suppress emotional vulnerability and are more likely to externalise distress through behavioural problems, substance use, or aggression [62]. It is important to consider that gender roles vary significantly across different cultural contexts, and this can influence how mental health problems develop. Furthermore, most of the available studies come from high-income countries (mainly Western countries), so some dynamics may differ in other cultural contexts.

This gendered emotional conditioning may also contribute to disparities in help-seeking behaviours and the recognition of mental health needs. Girls are more likely to access psychological support, while boys often delay or avoid seeking help due to stigma and cultural notions of masculinity [63].

Other systemic barriers include the lack of gender-sensitive educational and mental health programmes in schools, which often fail to consider the different emotional processing and coping strategies of boys and girls. Additionally, interventions and assessments may still reflect gender biases, limiting their responsiveness to diverse psychosocial needs.

In sum, a comprehensive understanding of adolescent mental health must address not only biological and psychological differences but also the cultural scripts and social inequities that shape the lived experiences of young males and females. Overcoming these barriers requires multi-level action, including gender-inclusive education, clinician training, and culturally informed prevention strategies.

### 4.2. Limitations of the Literature and Future Directions

Many studies have methodological limitations, such as lack of long-term follow-up or geographically or culturally limited samples. Furthermore, gender differences in many psychiatric disorders remain poorly investigated, both in terms of onset and diagnostic criteria. Another limitation concerns the search strategy: although it was developed systematically, we acknowledge that keywords such as “psychiatric disorder” or “mental illness” were not included. The terms used were selected to reflect the main objective of the review, namely, to explore gender differences in adolescent psychiatric disorders. This choice may have limited the inclusion of some studies, with possible implications for the completeness of the evidence.

Another limitation concerns the poor representation of a sample with a population with different gender identities, as most of the literature focuses exclusively on samples with a cisgender population. The distribution of studies is also mainly concentrated in high-income countries, often neglecting the situation in diverse settings with limited resources. Future research should include more gender-sensitive diagnostic tools and also investigate underrepresented clinical groups and extend data collection to middle- and low-income countries in order to obtain a more comprehensive and representative picture.

## 5. Conclusions

This exploratory review has highlighted how psychiatric disorders in adolescence manifest themselves with marked gender differences, particularly in terms of prevalence, symptomatology and clinical pathways. Gender diversity and mental health problems stem from a complex interaction between biological, psychological and environmental factors during adolescence [64]. There is therefore a difference between girls and boys, but it is essential that mental health professionals adopt a personalised approach that is sensitive to cultural differences and takes into account not only biological sex but also gender identity and the adolescent’s social context.

One gap to highlight is that healthcare professionals need ongoing training on these issues in order to reduce the risk of underdiagnosis or late diagnosis, especially in boys with masked internalised symptoms or girls with underestimated externalised behaviours: for such boys, for example, multimodal approaches are recommended, including psychoeducation, behavioural interventions and the involvement of school and family; for girls, it may be useful to supplement psychotherapy with interventions focused on emotional regulation, self-esteem, and family support.

In the editorial “Placing research in context—what participant and study characteristics should be routinely reported in studies of child and adolescent mental health?”, Megan R. Gunnar emphasises the importance of transparently reporting key data such as age, gender, socioeconomic background, ethnicity, and minority status in order to better understand the factors that influence the onset and evolution of psychiatric problems.

In summary, adopting a multidisciplinary, gender-sensitive, evidence-based approach is a fundamental step towards improving timeliness, diagnostic accuracy and therapeutic effectiveness in adolescent mental health [65].

## Data Availability

No new data were created or analysed in this study.
